# Supplementary material for: Utility of Single Items within the Suicidal Behaviors Questionnaire-Revised (SBQ-R): A Bayesian Network Approach and Relative Importance Analysis
Source: Behav Sci (Basel). 2024 May 14;14(5):410. doi: 10.3390/bs14050410 (PMC11117767; doi:10.3390/bs14050410)
Supplement: Supplementary file 1 [file behavsci-14-00410-s001.zip › behavsci-2906370-supplementary.pdf]

Figure S1

Extended Results from Table 2: Set of Conditional Probability Tables for the U.S. Undergraduate Sample (*N = 1,160*)

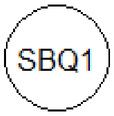

	**SBQ1** (History of suicidal thoughts and attempts)			
	Never	Passing thought	Had a plan	Attempted
	0.48	0.29	0.16	0.07

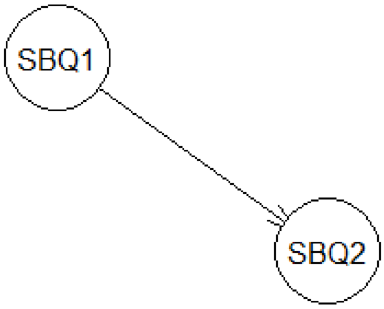

**SBQ2** (frequency of suicidal ideation)	**SBQ1** (History of suicidal thoughts and attempts)			
	Never	Passing thought	Had a plan	Attempted
Never	0.99	0.40	0.20	0.19
1 time	0.01	0.38	0.27	0.19
2 times	0	0.16	0.26	0.21
3-4 times	0	0.04	0.16	0.18
5 or more times	0	0.02	0.11	0.22

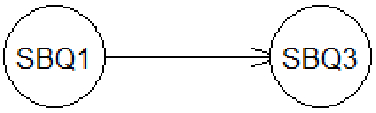

**SBQ3** (communication of intent to die by suicide)	**SBQ1** (History of suicidal thoughts and attempts)			
	Never	Passing thought	Had a plan	Attempted
No	0.98	0.73	0.44	0.29
At one time	0.02	0.25	0.43	0.35
More than once	0.01	0.02	0.13	0.36

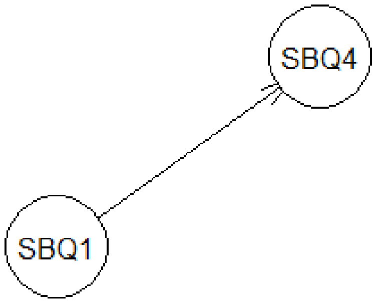

**SBQ4** (self-reported likelihood of a suicide attempt)	**SBQ1** (History of suicidal thoughts and attempts)			
	Never	Passing thought	Had a plan	Attempted
Never	0.89	0.58	0.19	0.19
No chance at all	0.08	0.24	0.16	0.13
Rather unlikely	0.03	0.15	0.39	0.23
Unlikely	0	0.02	0.15	0.13
Likely	0	0	0.08	0.27
Rather likely	0	0	0.02	0.03
Very likely	0	0	0	0.01

Figure S2

Extended Results from Table 3: Set of Conditional Probability Tables for the Chinese Undergraduate Sample (*N* = *1,141*)

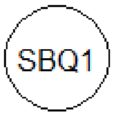

	**SBQ1 (History of suicidal thoughts and attempts)**			
	Never	Passing thought	Had a plan	Attempted
	0.70	0.22	0.06	0.02

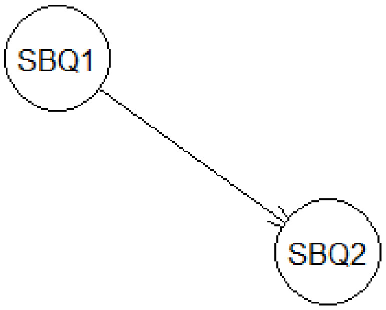

**SBQ2** (frequency of suicidal ideation)	**SBQ1 (History of suicidal thoughts and attempts)**			
	Never	Passing thought	Had a plan	Attempted
Never	0.97	0.51	0.29	0.32
1 time	0.02	0.40	0.32	0.18
2 times	0	0.06	0.25	0.23
3-4 times	0	0.04	0.12	0.14
5 or more times	0	0	0.03	0.14

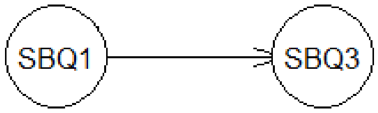

**SBQ3** (communication of intent to die by suicide)	**SBQ1 (History of suicidal thoughts and attempts)**			
	Never	Passing thought	Had a plan	Attempted
No	0.98	0.72	0.41	0.23
At one time	0.02	0.26	0.47	0.45
More than once	0	0.02	0.12	0.32

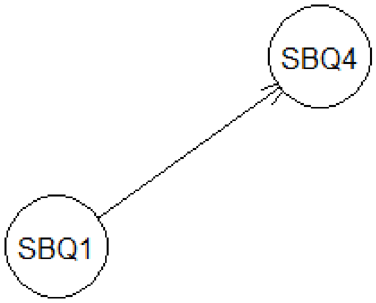

**SBQ4** (self-reported likelihood of a suicide attempt)	**SBQ1 (History of suicidal thoughts and attempts)**			
	Never	Passing thought	Had a plan	Attempted
Never	0.79	0.49	0.16	0.23
No chance at all	0.15	0.20	0.19	0
Rather unlikely	0.03	0.15	0.11	0.09
Unlikely	0.02	0.08	0.21	0.09
Likely	0.01	0.08	0.26	0.45
Rather likely	0	0	0.07	0.09
Very likely	0	0	0	0.05
